# CKD-related impairment in humoral and cellular immune response and potential correlation with long COVID-19: a systematic review

**DOI:** 10.3389/fimmu.2025.1690298

**Published:** 2025-10-29

**Authors:** Dali Wang, Fenghua Zhang

**Affiliations:** ^1^ Center for Clinical and Translational Medicine, Shanghai University of Medicine and Health Sciences, Shanghai, China; ^2^ Department of Laboratory Medicine, Shanghai University of Medicine & Health Sciences Affiliated Zhoupu Hospital, Shanghai, China

**Keywords:** COVID-19, chronic kidney disease, humoral immune response, cellular immune response, immune dysfunction

## Abstract

**Introduction:**

Patients with chronic kidney disease (CKD) are at high risk of morbidity and mortality from SARS-CoV-2 infection (COVID-19). However, their immune response to vaccination may vary among individuals. The purpose of this review was to identify characteristics of alterations in humoral and cellular immune responses to the vaccination, and to provide insights into their immune dysfunctions for a better care of acute COVID-19 and prevention of long COVID-19.

**Methods:**

PubMed, Embase, Scopus, Web of science and Cochrane Central were systematically searched. Eligible publications included clinical studies reporting immune response to COVID-19 vaccination in CKD patients without dialysis or KT, CKD patients undergoing dialysis, as well as CKD patients with KT. Demographics, measurements and results of their humoral and cellular response were evaluated, and the quality of studies were assessed using the Joanna Briggs Institute (JBI) critical appraisal tool and the Newcastle-Ottawa quality assessment scale (NOS).

**Results:**

A total of 31 eligible studies were identified. A decreased proportion of patients with KT showed anti-S IgG positivity after the 2^nd^ (67%) and 3^rd^ (56.6%) dose of vaccination. Similarly, a decreased proportion of these patients presented S-specific T-cell response after the 2^nd^ (17.7%) and 3^rd^ (12.9%) dose. Though lower anti-S IgG titers in patients with CKD or on dialysis, as well as T-cell response in patients on dialysis were reported to be lower after the 2^nd^ or 3^rd^ dose of vaccination, conflicting results were reported by other studies. Limited studies on correlated change between humoral and cellular immune response revealed a low rate of co-presence of the two in patients with dialysis, though antibody level was correlated with rate of cellular response, while no such correlation was revealed in patients with KT.

**Conclusion:**

The study provides crucial information on features of humoral and cellular immune responses to COVID-19 vaccinations in CKD patients, and suggests possible directions for strategy of management such as antibody monitoring, additional booster dose or immunomodulatory therapies not only for acute COVID-19 but also for long COVID-19.

## 1 Introduction

Chronic kidney disease (CKD) is considered as a risk factor for severe acute respiratory syndrome coronavirus 2 (SARS-CoV-2) infection (1), and patients with CKD, with or without interventions like dialysis or kidney transplantation (KT), are at high risk of morbidity and mortality due to SARS-CoV-2 infection. Specifically, CKD patients who depend on dialysis encountered the highest risk of death from COVID-19 within the population, with a 28-day probability of death being 25% for patients undergoing hemodialysis (HD) and 33.5% for those that were admitted to hospitals before initiation of population vaccinations, as reported by the European Renal Association COVID-19 Database (ERACODA) report (2). In addition, the mortality rates in dialysis patients exceeded 20% (3), which was approximately 10 times higher among HD patients (4), probably be due to the impaired immunity associated with their primary disease, presence of more comorbidities and utilization of immunosuppressive drugs (5).

Long COVID-19 presents a variety of symptoms that persist for 3 months or longer after SARS-CoV-2 infection. Investigations on long COVID-19 revealed that survivors presented a significant decrease in estimated glomerular filtration rate (eGFR) in observations for up to one year (6–8). Together, the high vulnerability to COVID-19 in CKD patients highlight the necessity of efficient prevention for these patients.

Vaccination has been considered as an efficient way of protecting individuals from COVID-19, particularly for the severe type. Importantly, previous study showed that vaccination either before or after SARS-CoV-2 infection was associated with reduced risk of long COVID-19 (9). However, its use was reported by some to have lower protection rates and special potential risks in populations such as CKD, including those undergoing HD, peritoneal dialysis (PD) or kidney transplant recipients (KTRs), while conflicting results were present and requires further validation. This is mainly attributed to the impaired immune response in those patients (10), which may work in two ways. CKD is associated with both immune activation and deficiency. Vaccination-induced immunity is based on adaptive immune response, which includes B cell-mediated response (humoral immunity) and T cell-mediated response (cellular immunity). Decrease in total number of B cells is associated with GFR reduction (11). In addition, both the number of naïve T cells and T cell subset distribution are affected in patients with CKD (12). Observation on effects of non-COVID vaccinations revealed that CKD patients tend to have a reduced immune response to vaccination (13), as it is marked by chronic inflammation and immune dysfunction that usually results in lower rates of seroconversion, lower antibody levels, and a less sustained humoral response to vaccination compared with the general population (14, 15). Consequently, a need for higher vaccine dosage to target immunogenicity in these patients was frequently encountered (16). On the other hand, renal events may occur in patients with a strong immune response due to immunological dysregulation in patients with glomerulonephritis and nephrotic syndrome.

Utilization of non-COVID-19 vaccines has been previously reported to be associated with development of nephritis, such as minimal change disease, membranous nephropathy, and vasculitis (17). New onset or relapse of glomerulonephritis and nephrotic syndrome have also been reported after COVID-19 vaccination (18), although its incidence and relevance remain unclear.

The present review evaluated and summarized current studies assessing humoral and cellular response to COVID-19 vaccination in CKD patients without dialysis or KT, CKD patients undergoing dialysis, as well as CKD patients with KT, aiming to identify features of changes in both types of immune response to the vaccination, evaluated differences in changes among these conditions and healthy controls, as well as correlations between humoral and cellular responses to COVID-19 vaccination in CKD, dialysis and KT, to provide an insight to their immune dysfunctions for a better care of acute COVID-19 and prevention of long COVID-19.

## 2 Methods

### 2.1 Searching strategies

Literature searching was performed on databases, which included PubMed, Embase, Scopus, Web of science and Cochrane Central, using searching terms (‘long COVID’ OR ‘post-COVID condition’ OR ‘post-COVID-19 condition’ OR ‘post-COVID-19 syndrome’ OR ‘post-acute COVID-19’ OR ‘chronic COVID-19’ OR ‘ongoing symptomatic COVID-19’) AND (‘kidney’ OR ‘renal’ OR ‘nephropathy’ OR ‘membranous’ OR ‘MN’ OR ‘nephritis’ OR ‘vasculitis’ OR ‘glomerular’ OR ‘glomerulopathy’ OR ‘glomerulonephritis’) to identify literatures published anytime until 9 February, 2025 in English.

### 2.2 Selection process and eligibility criteria

**Figure 1:**
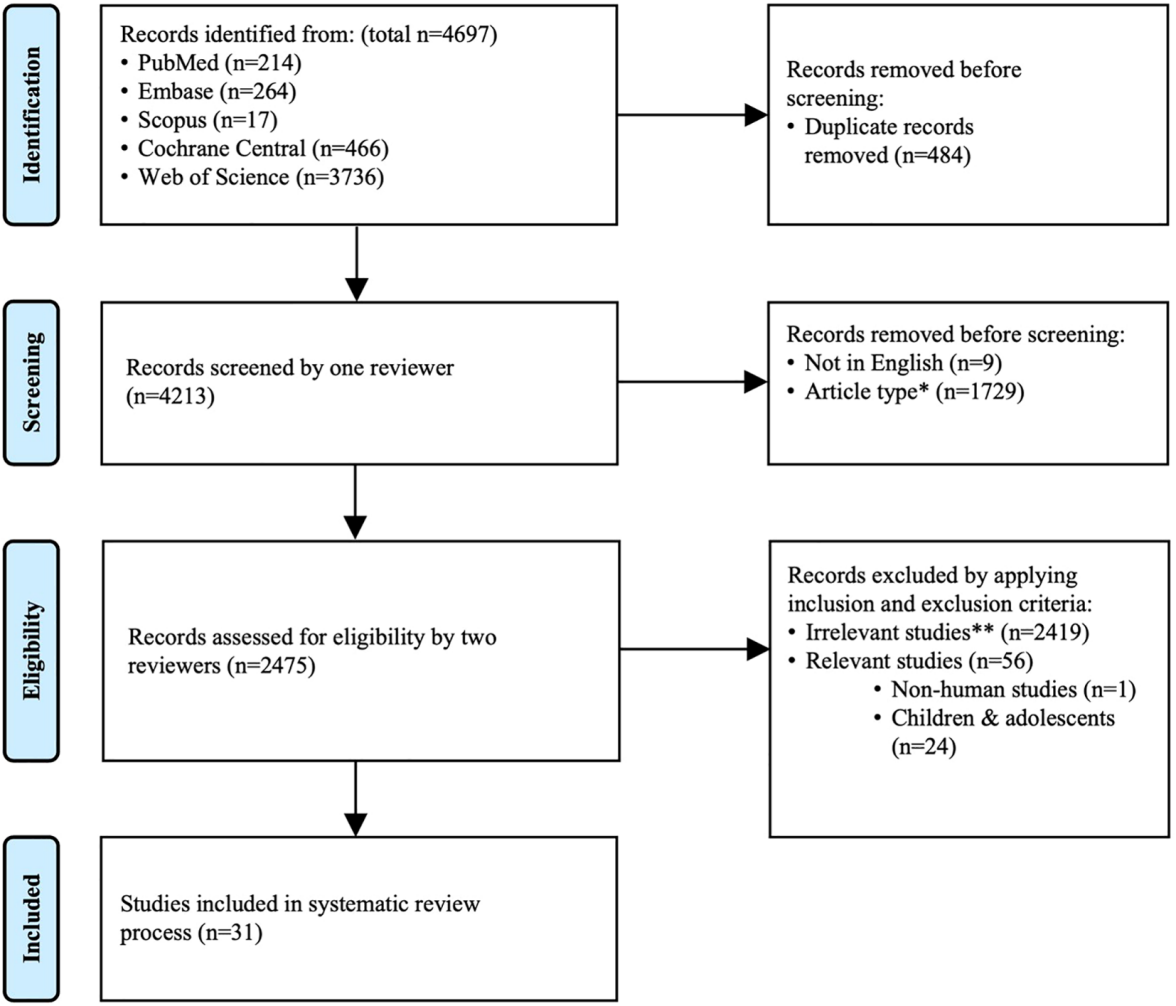
PRISMA flow diagram. The present review was reported according to PRISMA. *Excluded article types include review and perspective, systematic review and meta-analysis, case report and case series, comment, conference paper, book chapter, letter and response, correction and erratum, retraction, editorial, guideline and consensus, protocol, rationale and design, survey, publication in other languages, as well as other miscellaneous article types. **Irrelevant studies are those that met exclusion criteria.

A total of 4697 records were identified, and searching results were imported to EndNote 20. Duplications were removed, and undesired article types including reviews and perspectives, systematic review and meta-analysis, case reports and series, comments, conference papers, book chapters, letters and response, correction and erratum, retractions, editorials, guideline and consensus, protocol, rationale and designs, surveys, publications in other languages, as well as other miscellaneous article types, were excluded. Subsequently, titles and abstracts were further reviewed to exclude irrelevant studies, which included studies not related to the topics or only partially related to the topics, or studies performed on other species or on individuals under 18 years of age. In addition, studies with full text unavailable were also excluded. Studies analyzing changes of immune response following COVID-19 vaccination in patients with CKD of various etiologies, patients on dialysis or underwent KT were included. The present systematic review was reported according to the Preferred Reporting Items for Systematic Reviews and Meta-analysis (PRISMA) (**Figure 1**).

### 2.3 Data extraction

Demographic information such as age, gender, kidney-related conditions and etiologies, as well as other information including sample size, previous COVID-19 infection, etiologies of kidney disease, types of vaccination and doses administrated, humoral response measurements, cellular response measurements, and main results were extracted using a self-developed standardized form. The mean age and standard deviation were calculated when the relevant information in subgroups was available.

### 2.4 Quality assessment

The risk of bias for cohort studies were assessed using the Joanna Briggs Institute (JBI) critical appraisal tool for cohort studies, and the risk of bias for case control study was evaluated using the Newcastle-Ottawa quality assessment scale (NOS). The JBI critical appraisal for cohort study was consist of 11 questions, and a response of “Y” for “yes”, “N” for ‘no’, “U” for ‘unclear’ or “NA” for ‘not applicable’ was marked for each question. Studies with less than 1 “N” were considered of presenting high quality, studies with no more than 2 “N” were considered of presenting moderate quality, while studies with more than 2 “N” were considered of presenting low quality. The NOS assessed domain of “selection”, “comparability”, as well as “exposure”, and a maximum of 4 stars, 2 stars and 4 stars could be achieved for each domain, respectively. Higher number of total stars indicate lower risk of bias.

## 3 Results

### 3.1 Study characteristics and demographics

**Figure 2:**
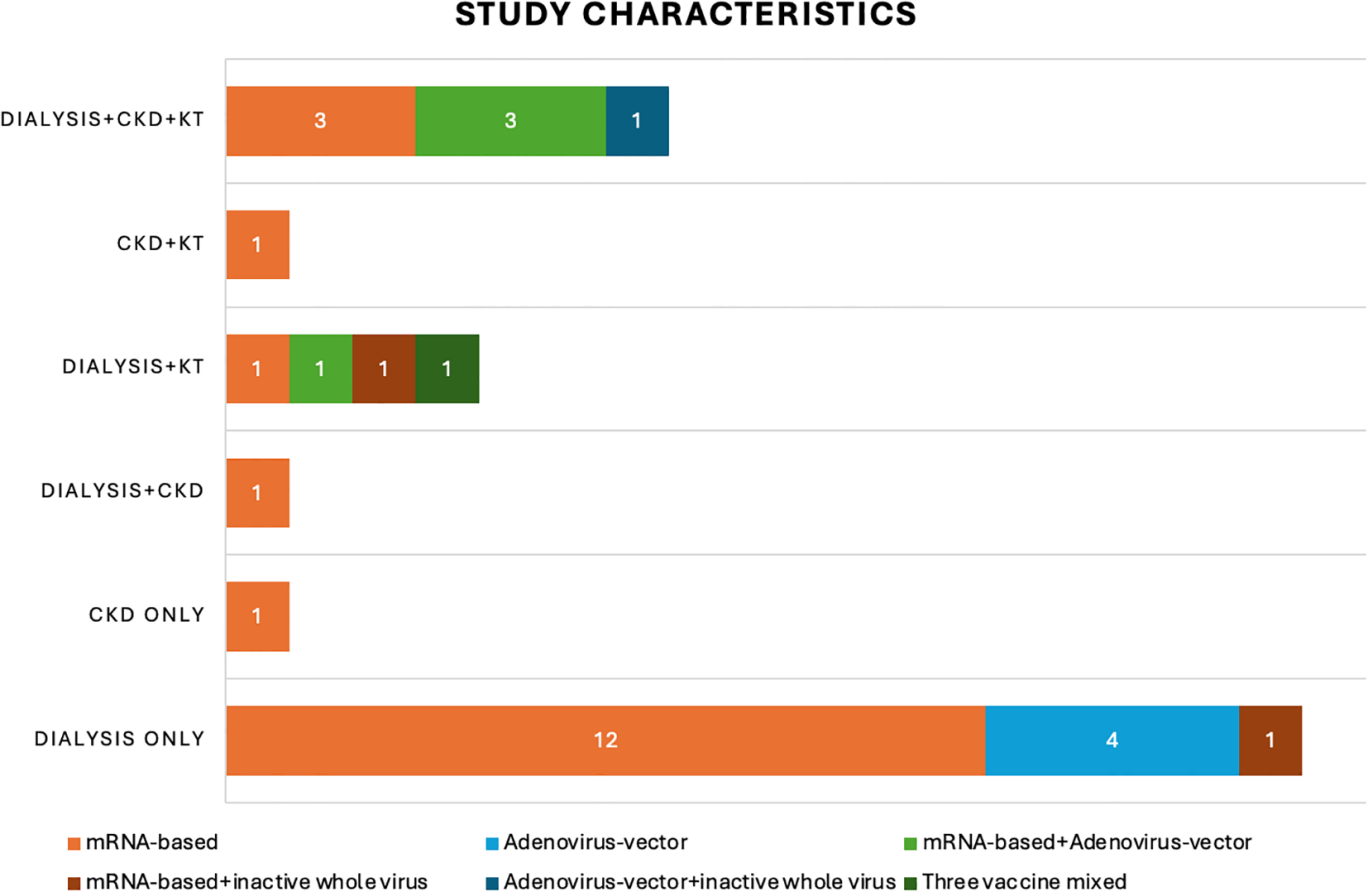
Study characteristics. This bar chart shows the number of recruited studies that fall into each categories: chronic kidney disease (CKD), dialysis, kidney transplant (KT) or mixed etiologies. The different colors of the bars indicate the number of studies using varied vaccination strategies in these 31 recruited studies: mRNA-based vaccine only (orange), adenovirus-vector vaccine only (light blue); mRNA-based vaccine + adenovirus-vector vaccine (light green); mRNA-based vaccine + inactive whole virus vaccine (red); adenovirus-vector vaccine + inactive whole virus vaccine (dark blue); three types of vaccines mixed (dark green).

A total of 31 publications have been identified as eligible studies (**Figure 2**), and the demographics, humoral response and cellular response extracted from these eligible studies are shown in **Table 1**. All studies that provided information on etiologies of CKD include diabetic nephropathy, which constitute 5.9-30.3% of the cohorts (21, 23, 25, 34, 40, 41, 43). Glomerulonephritis was frequently reported by 5 studies, which constituted 10-40% of the population (21, 23, 25, 34, 43). Interstitial nephritis were reported by three studies, constituted 4.6-10% of the cohort (25, 34, 43), and vascular nephropathy constituted 19-20.8% of CKD, as reported by 3 studies (21, 25, 43). Congenital kidney diseases, familial/hereditary diseases and pyelonephritis constituted 3.9-4.2%, 16.4-16.7% and 0.7-1.4% of CKD, respectively, as reported by two studies (25, 43). In addition, nephroangioesclerosis (30%) (34), hypertensive (15.4%) (23), polycystic kidney disease (20%) (34) were also reported. Furthermore, secondary kidney diseases constituted 2.6-4.2% of CKD, as reported by 2 studies (25, 43). CKD with etiology marked as “other” or “unknown” were frequently reported by studies, constituting a considerable proportion of CKD. Specifically, “other etiology” constituted 3.3-40.3% of CKD (21, 23, 25, 34, 43), and “unknown etiology” constituted 2-20% of CKD (21, 25, 34, 43) (**Table 1**).

**Table 1:** Demographics, humoral response and cellular response from eligible studies.

Ref	Grouping and sample size	Age (mean ± SD or median)	Gender (%F)	Vaccination type	Total dose	Etiology	Humoral response measurement	Cellular response measurement	Main results
(19)	• Dialysis (n=175) • KT (n=252) • CG (n=71)	56.4 ± 14.4	40	mRNA-based	2	/	Anti-SARS-CoV-2 S1/S2 IgG	/	• Anti-S1/S2 IgG 3 months after vaccination:- (+) in 79% dialysis, 42% KT and 100% CG • Anti-S1/S2 IgG after infection:- (+) in 94% dialysis, 94% KT and 100% CG • Predictors of non-response:- Old age, diabetes, history of cancer, low lymphocyte count, low Vitamin D • Factors associated with lower level of IgG in dialysis- Dialysis modality, high serum ferritin levels • Factors associated with lower level of IgG in KT- Hypertension, higher calcineurin, mTOR inhibitor drugs
(20)	• IN-Ds (n=109) • PI-Ds (n=32) • CG (n=20)	• IN-D: 69 • PI-D: 65 • CG: 53	35.4	mRNA-based	3	/	SARS-CoV-2 anti-S IgG	/	• Anti-S IgG titer at 3 months after 2^nd^ dose:- IN-D < CG < PI-D• Anti-S IgG titer at 6 months after 2^nd^ dose- IN-D < CG < PI-D • Reduction of IgG titer at 3 and 6 months after 2^nd^ dose:- IN-D: 82.9%; 93.03%- PI-D: 73.4%; 93.36%- CG: 75.5%; 88.8% • Anti-S IgG seroconversion at 3 and 6 months after 2^nd^ dose:- Dialysis: 82.6%; 67.9%- CG: 11%; 95% • Anti-S IgG protective titer at 3 and 6 months after 2^nd^ dose:- Dialysis: 46.6%; 23.8%- CG: 95%; 70%
(21)	• CKD (n=160) • HD (n=206) • KT (n=216)	• CKD: 63.1• HD: 69.5• KT: 59.9	• CKD: 41%• HD: 32%• KT: 32%	mRNA-based	2	• Diabetic nephropathy (9%, 22%, 13%)• Glomerulonephritis (40%, 17%, 33%)• Others (31%, 27%, 36%)• Unknown (2%, 11%, 5%)• Vascular nephropathy (19%, 23%, 13%)	• SARS-CoV-2 IgG II• SARS-CoV-2 S1/S2 IgG	/	• No response at 3 months after 1^st^ dose- CKD: 12.5%- KT: 50%• IgG titers- ↑ in all groupsCKD > HD, KT • Factors associated with non-response- CKD: treatment with rituximab- HD: renal transplant *in situ*; use of calcineurin inhibitors- KT: age, use of mycophenolic acid, glucocorticoids
(22)	• HD and PD (n= 315)	65.5 ± 12.38	38.6%	Adenoviral vector-based	2	/	Anti-S-RBD	CD19, CD3, CD4, CD8, CD56, CXCR3, CD69, IgG	• Positive Ab response:- 2 weeks after 1^st^ dose:37.66%- 10 weeks after 1^st^ dose: 65.58%- 4 weeks after 2^nd^ dose: 94.16% • Features for immune naive patients- ↓ early active B cells- ↓ proliferative B cells- ↑ cNK
(23)	• CKD (n=285)	67	42%	mRNA-based	3	/	• SARS-CoV-2 anti-S• SARS-CoV-2 anti-RBD• SARS-CoV-2 anti-NP	/	• Anti-S and anti-RBDPeak at 2 months after 3^rd^ dose• Seropositivity rate over 9 monthsAnti-S: 93%Anti-RBD: 85% • CKD on immunosuppressive treatmentLess likely to mount a robust anti-S response • Ab level over timeMore pronounced decline in older patients
(24)	• KT (n=113) • Dialysis (n=108)	49.9	43.9%	• Inactive whole-virus vaccine •Adenovirus vector vaccine • mRNA-based	1 or 2 doses	• Undetermined: 32.6% •Glomerulonephritis: 26.7% • DM: 18.1% • PKD: 9% • HTN: 8.1% • Urologic: 5.4%	• Anti-S-RBD IgG • Neutralizing Ab	/	• IgG titer- KT < dialysis• Neutralizing antibodies- (-) between groups
(25)	• CG (n=93) • CKD (n=81) • Dialysis (n=77) • KT (n=141)	CG57.7 ± 13.6CKD59.4 ± 13.1Dialysis60.3 ± 14.8KT: 56.4 ± 12.8	CG: 58.1%CKD: 33.3%Dialysis: 32.55%KT: 48.6%	mRNA-based	2	• GlomerulonephritisCKD: 13.9Dialysis: 13.2%KT: 19.4%• PyelonephritisCKD: 1.4%Dialysis: 0%KT: 1.6%• Interstitial nephritisCKD: 5.6%Dialysis: 2.9%KT: 5.6%• Familial/hereditary renal diseasesCKD: 16.7%Dialysis: 19.1%KT: 25%• Congenital diseasesCKD: 4.2%Dialysis: 1.5%KT: 5.6%• Vascular diseasesCKD: 20.8%Dialysis: 22.1%KT: 9.7%• Secondary glomerular/systemic diseaseCKD: 4.2%Dialysis: 5.9%KT: 5.6%• DMCKD: 6.9%Dialysis: 17.6%KT: 4.8%• OthersCKD: 23.6%Dialysis: 13.2%KT: 17.7% • UnknownCKD: 2.8Dialysis: 4.4%KT: 4.8%	• SARS-CoV-2 S1 IgG	• SARS-CoV-2-specific IFN-*γ* T cell response	• SARS-CoV-2-specific T cell response- ↓ in 43.4% dialysis- ↓ in 42.6% KT- ↓ in 70% CKD- ↓ in 76% CG • Use of calcineurin inhibitor ⇔↓ T cell response in KT • Co-presence of humoral and T-cell response- 76.1% CG- 70.4% CKD- 54.5% Dialysis- 27.9% KT • Humoral and cellular non-responder ⇔ MMF use, lower lymphocyte count, and ↓ eGFR
(26)	• CKD (n=18) • Vasculitis (n=7) • KT (n=17)	54 ± 13	47.6%	mRNA-based	2	/	SARS-CoV-2 S1 IgGNeutralization	• Specific T-cell response	• Neutralizing antibody titers↑ after vaccine in CKD• T-cell response↑ after vaccine in CKD • T-cell response(-) in KT
(27)	• Dialysis (n=121) • CG (n=104)	61.4 ± 13.1	48.9%	mRNA-based	2	/	• Anti-S IgG	/	• Ab titer- ↑ at 6–8 months after 3^rd^ dose • Ab titer- (-) between groups at 4–8 months after 2^nd^ dose • Non-response after 2^nd^ dose- Dialysis: 9.09%- CG: 3.85%
(28)	• HD (n=281)	68	39.9%	mRNA-based	2	/	SARS-CoV-2 anti-S IgG	specific T-cell response	• Cellular immunity- ↑ in patients with pre-vaccine infection- Depend on albumin level • Factors influencing Ab level after vaccination- Previous infection, age, NLR, absolute neutrophil count, Hb level
(29)	• HD (n=85) • PD (n=24)	64 ± 14	31.3%	mRNA-based	2 or 3	/	SARS-CoV-2 IgG	/	• Seroconversion rate • Predictive factor for non-response- Immunosuppressive therapy
(30)	• CG (n=35) • CKD G4(n=48) • CKD G5(n=35) • HD (n=70)	74.9 ± 8.4	36.7%	mRNA-based	2	/	SARS-CoV-2 IgG	/	• Anti-S IgG positivity after 2^nd^ dose- 100% in G4 and G5- 98.5% in HD • Median value of anti-S IgG- ↓ more in HD than CKD G4 and G5- (-) between CKD G4 and G5- ↓ at 6 months compared to 1 month after 2^nd^ dose in HD
(31)	• HD (n=50) • 7-15w follow up (n=50) • 3 month follow up (n=40)	69.9 ± 13.4	35.8%	mRNA-based	3	• DM- 46%• Nefrosclerosis- 30%• Autosomal PKD- 8%• Chronic glomerulonephritis- 16%• Vascular/anti-GBM-nephropathy- 8%	SARS-CoV-2 anti-S IgG	T-cell activity	• Anti-S IgG at 7-15w after 2^nd^ dose- Positivity: 88% • Anti-S IgG at 3 month after 3^rd^ dose- Positivity: 95%↑ than 7-15w • Positivity of T-cell activity- 6–8 month after 2^nd^ dose: 55%- 3w after 3^rd^ dose: 85%- 3 months after 3^rd^ dose: 71%
(32)	• HD (n=185) • CG (n=109)	62 ± 12	50%	mRNA-based	2	/	SARS-CoV-2 anti-S1 IgG	/	• Anti-S IgG positivity- 97.6% in HD- 100% in CG • Factors associated with low response- Old age, low BMI, low Cr index, low nPCR, low GNRI, low lymphocyte count, use of steroid, complications related to blood disorders
(33)	• HD (n=167) • CG (n=100)	• HD: 70 • CG: 54	50.2%	mRNA-based	3	/	SARS-CoV-2 anti-S IgG	/	• Anti-S IgG positivity at 2w after 2^nd^ doseHD: 97.6%CG: 100% • Anti-S IgG positivity at 2w after 3^rd^ doseHD: 99.4%CG: 100% • Factors involved in low response after 2^nd^ doseOld age, low BMI, low Cr index, low nPCR, low GNRI, low lymphocyte count, use of steroid, complications related to blood disorders
(34)	• HD (n=52) • PD (n=14) • Scheduled KT (n=30) • ACKD (n=30) • CG (n=18)	• HD: 72• PD: 69• Scheduled KT: 59• ACKD: 66• CG: 63	• HD: 32.7%• PD: 21.4%•Scheduled KT: 26.7%•ACKD: 36.7%• CG: 72.2%	• mRNA-based •adenovirus vector vaccine	2 or as instructed	•Nephroangioesclerosis- HD: 15.4%- PD: 35.7%- Scheduled KT: 3.3%- ACKD: 30%• DM- HD: 15.4%- PD: 35.7%- Scheduled KT: 16.7%- ACKD: 6.7%• Chronic interstitial nephritis- HD: 5.8%- PD: 0%- Scheduled KT: 10%- ACKD: 10%• Cystic disease- HD: 9.6%- PD: 0%- Scheduled KT: 0%- ACKD: 20%• Urologic- HD: 1.9%- PD: 0%- Scheduled KT: 6.7%- ACKD: 0%• Glomerulonephritis- HD: 17.3%- PD: 14.3%- Scheduled KT: 43.4%- ACKD: 10%• Unknown- HD: 32.7%- PD: 14.3%- Scheduled KT: 16.7%- ACKD: 20%• Other- HD: 1.9%- PD: 0%- Scheduled KT: 3.3%- ACKD: 3.3%	• Anti-S IgG	• T-cell response	• Anti-S IgG at 15 days after 2^nd^ dose- HD: 95%- PD: 93%- Scheduled KT: 67%- ACKD: 96%- CG: 81% • Anti-S IgG at 3 months after 2^nd^ dose- HD: 98%- PD: 100%- Scheduled KT: 75%- ACKD: 100%- CG: 100% • T-cell response at 15 days after 2^nd^ dose- PD: 93%- HD: 70%- Scheduled KT: 84%- ACKD: 80%- CG: 67% • T-cell response at 3 months after 2^nd^ dose- HD: 91%- PD: 100%- Scheduled KT: 96%- ACKD: 89%- CG: 89%
(35)	• HD (n=22) • CG (n=28)	55.7 ± 12.3	57.9%	Adenovirus-based	2	Glumerulonephritis: 27%HTN: 18%DM: 5%Hereditary kidney disease: 36%Other/miscellaneous: 14%	• SARS-CoV-2-anti-S1 IgG	• T-cell response	• Anti-S1 IgG at 6 months compared to 1 month after 2^nd^ dose- ↓ in both groups • T-cell positivity- HD: 67%- CG: 48% • T-spot counts at 6 months compared to 1 month after 2^nd^ dose- ↓ in CG
(36)	• HD (n=21) • CG (n=15)	58.9 ± 13.1	52.8%	mRNA-based	2	/	• SARS-CoV-2 anti-S IgG	/	• Anti-S IgGHD < CG
(37)	• KT (n=52) • HD (n=48) • CG (n=15)	• KT53.7 ± 12.7 • HD68.3 ± 13.9 • CG: 36	• KT: 32.7% • HD: 25% • CG: 67%	• First 2 dosesmRNA-based or inactive whole-virus vaccine • Booster dosemRNA-based	2 + 1	• DM - KT: 13.5% - HD: 31.2% • Unknown - KT: 25% - HD: 31.7% • Glomerular - KT: 34.6% - HD: 16.7% • Congenital/genetic - KT: 21.1% - HD: 8.3% • Others - KT: 5.8% - HD: 2.1%	• SARS-CoV-2 anti-S IgG	• T-cell activity •Polyfunctional CD4+ and CD8+ T cells • Memory T cells	• Anti-S IgG at 4 months after 2^nd^ dose- KT < HD < CG • Anti-S IgG at 1 month after 3^rd^ dose- ↑ more in KT than in HD • Neutralizing Ab after 3^rd^ dose- KT < HD and CG • T-cell response after 2^nd^ dose- CKD < CG • T-cell response and humoral response after 2^nd^ dose- T-cell response: 75% KT- Humoral response: 49% KT • Triple positive CD4+ polyfunctional T cells after 2^nd^ dose- ↑ more in KT than HD • Double and triple positive CD4+ T cells after 3^rd^ dose- ↑ more in KT than HD and CG • CD4+ and CD8+ Memory T cell response after 3^rd^ dose- ↑ in all groups
(38)	• Dialysis Vaccinated (n=321) • Dialysis Recovered from COVID-19 (n=183)	•Vaccinated: 67 •Recovered: 70	41.1%	mRNA-based	2	• DMVaccinated: 15.3%Recovered: 45.4%• HTNVaccinated: 8.4%Recovered: 20.8%• PKDVaccinated: 4.0%Recovered: 12.0%• GlomerulonephritisVaccinated: 6.9%Recovered: 31.7%• Chronic pyelonephritisVaccinated: 5.9%Recovered: 13.1%• OthersVaccinated: 16.5%Recovered: 52.5%	• SARS-CoV-2 anti-S IgG	/	• Age ⇎ anti-S IgG
(39)	• HD (n=38)	49	47.4%	• Inactive whole-virus • mRNA-based	2 + 1	/	• SARS-CoV-2 anti-S-RBD IgG	/	• GMT of anti-S-RBD - ↑ at 8 months after 2^nd^ dose - ↑ at 1 month after 3^rd^ dose • Median inhibition rate of Nabs - (-) between after 2^nd^ dose and after 3^rd^ dose
(40)	• KT (n=283) • HD (n=1116) • PD (n=171) • CKD (n=176)	63.67 ± 13.28	37.5%	• mRNA-based • adenovirus vector-based	/	• DMKT: 4%HD: 25%PD: 22%CKD: 26%	• SARS-CoV-2 anti-S IgG	/	• Anti-S IgG positivity at 1 month after vaccine - KT: 79% - HD: 98% - PD: 99% - CKD: 100%
(41)	• CKD (n=109) • HD (n=1517) • PD (n=164) • KT (n=396)	66.5	36.6%	• Regular dose - mRNA-based -adenovirus vector-based • Booster dosemRNA-based	•Regular dose2 •Booster dose1 or 2	• DM - CKD: 30.3% - HD: 24.8% - PD: 18.9% - KT: 5.1%	• SARS-CoV-2 anti-S IgG	/	• Anti-S IgG titers - ↑ after 4^th^ dose in HD and CKD • Seroconversion for previously negative patients - 72%
(42)	• HD (n=72) • CG (n=72)	43.2 ± 7.9	43.1%	•Adenovirus vector-based	1	• DM: 25% •Glomerulonephritis: 19.4% • Chronic interstitial nephritis: 8.3% • Obstructive uropathy: 6.9% • Hypertensive nephrosclerosis: 4.2% • Autosomal dominant PKD: 2.8% • Unknown: 33.3%	• SARS-CoV-2 anti-S RBD IgG	/	• Anti-S-RBD IgG positivityHD: 88.9%CG: 100% • Age and sodium level ⇎ ↓ Ab titer • Age ⇎ non-responders
(43)	• CKD (n=152) • Dialysis (n=145) • KT (n=267) • CG (n=181)	58.3 ± 13.6	44.4%	mRNA-based	2	• Glomerulonephritis - CKD: 11.8% - Dialysis: 9.7% - KT: 19.9% • Pyelonephritis - CKD: 0.7% - Dialysis: 0.7% - KT: 1.5% • Interstitial nephritis - CKD: 4.6% - Dialysis: 2.8% - KT: 3.4% • Familial/hereditary renal diseases - CKD: 16.4% - Dialysis: 13.1% - KT: 19.1% • Congenital diseases - CKD: 3.9% - Dialysis: 3.4% - KT: 6.7% • Vascular diseases - CKD: 20.4% - Dialysis: 18.6% - KT: 9.7% • Secondary glomerular/systemic disease - CKD: 2.6% - Dialysis: 4.8% - KT: 4.5% • DM - CKD: 5.9% - Dialysis: 14.5% - KT: 3.7% • Others - CKD: 19.1% - Dialysis: 16.6% - KT: 14.6% • Unknown - CKD: 14.4% - Dialysis: 15.9% - KT: 16.8%	• SARS-CoV-2 anti-S1 IgG	• SARS-CoV-2-specific T-cell response	• Anti-S IgG positivity at 6 months after 2^nd^ dose - CKD: 98.7% - Dialysis: 95.1% - KT: 56.6% - CG: 100% - ↓ compared to 1 month after 2^nd^ dose • T-cell response at 1 month after 2^nd^ dose - CKD: 77.8% - Dialysis: 73.3% - KT: 17.7% - CG: 87.5% • T-cell response at 6 months after 2^nd^ dose - CKD: 59.4% - Dialysis: 52.6% - KT: 12.9% - CG: 75% - ↓ more in dialysis and KT than in CG • T-cell response ⇔ anti-S IgG at 1 month and 6 months after 2^nd^ dose
(44)	• HD (n=96)	36.70 ± 11.53	22.9%	•Adenovirus vector-based	2	/	• SARS-CoV-2 anti-S IgG	/	• Seronegative rate - 23.52% in 1 month after 1^st^ dose - 64.7% in 1 month after 2^nd^ dose • Non-responding rate - 35.29% at 1 month after 2^nd^ dose
(45)	• HD (n=81) • CG (n=34)	• HD: 69 • CG: 54.5	• HD: 41.98% • CG: 82.35%	mRNA-based	2	/	• SARS-CoV-2 IgG	• SARS-CoV-2-specific T-cell response • Cytokine measurements	• Diminished anti-S1 IgG at 3w after 2^nd^ doseMore in HD than in CG • Neutralization at 3w after 2^nd^ dose↓ more in HD than CG • T-cell responseLower in HD at 3w after 2^nd^ dose • Moderate correlation between T-cell response and B-cell response
(46)	• KT (n=30) • Dialysis (n=17)	• KT: 62 • Dialysis: 55	29.8%	• Adenovirus vector-based • mRNA-based	3	• DMKT: 33%Dialysis: 29%• GlomerulonephritisKT: 33%Dialysis: 47%• HTNKT: 7%Dialysis: 6%• OthersKT: 27%Dialysis: 18%	Anti-S RBD IgGSARS-CoV-2 NAb	CD4+ T cell countCD8+ T cell countNK cell countMonocyte countGranulocyte count	• Anti-S RBD IgG positivity - KT: 85.2% - Dialysis: 100% - KT < dialysis • Anti-NAbKT < dialysis • Predictors for poor serological response in KT - Vaccine type, higher mycophenolate dose, lower absolute B cell counts - Higher CD19+ B cell counts ⇔ seropositive response • Predictors for poor serological response in dialysis - Vaccine type, higher monocyte counts - Lower monocyte counts ⇔ seropositive response
(47)	• CKD (n=12) • HD (n=134) • CAPD (n=4) • KT (n=7) • CG (n=55)	54.8 ± 16.07	50.94%	•Adenovirus vector-based • Inactive whole-virus-based	2	/	• SARS-CoV-2 anti-S1/RBD IgG • Neutralizing Ab • Anti-neucleocapsid IgG	• T-cell response	• Seroconversion rate at 3 months after 2^nd^ dose - CKD: 100% - HD: 80.18% - CAPD: 0% - KT: 42.86% - CG: 92.31% • Anti-S IgG - Similar among CKD, HD and CG before and 3 months after 2^nd^ dose - CAPD < HD and CG - Became (+) at 3 months after 2^nd^ dose in KT • NA level - Above protective level in all groups • T-cell response at 3 months after 2^nd^ dose - CKD < CG - Lowest in CAPD and KT
(48)	• HD (n=142) • CG (n=35)	• HD: 72 • CG: 46	45.9	mRNA-based	1 or 2	• DM: 44% • Ischemic nephropathy: 19% •Glomerulonephritis: 14%	• SARS-CoV-2 anti-S IgG • SARS-CoV-2 anti-RBD IgG • SARS-CoV-2 anti-NP IgG	/	• Seroconversion rate at 1 month after 1^st^ dose in HD patients received one doseAnti-S: 80%Anti-RBD: 55% • Seroconversion rate at 2w after 2^nd^ dose in HD patients received 2 dosesAnti-S: 96%Anti-RBD: 88%
(49)	• HD (n=194) • CG (n=103)	66.4 ± 11.3	37.2%	mRNA-based	2 + 1	/	• SARS-CoV-2 anti-S1 IgG • Neutralizing Ab titers	• T-cell response	• Anti-S IgG at 3w and 3 months after 3^rd^ doseHD > CG • Neutralizing Ab at 3w and 3 months after 3^rd^ doseHD > CG • T-SPOT at 3w and 3 months after 3^rd^ doseHD > CG

SD, standard deviation; KT, kidney transplantation; CG, control group; IN-D, infection-naïve dialysis patients; PI-D, previously infected dialysis patients; CKD, chronic kidney disease; cNK, cytotoxic natural killer; Ab, antibody; DM, diabetes mellitus; PKD, polycystic kidney disease; HTN, hypertension; MMF, mycophenolate mofetil; eGFR, estimated glomerular filtration rate; NLR, neutrophil to lymphocyte ratio; Hb, hemoglobin; HD, hemodialysis; PD, peritoneal dialysis; Cr, creatinine; BMI, body mass index; nPCR, normalized protein catabolic rate; GNRI, geriatric nutritional risk index; ACKD, advanced chronic kidney disease; GMT, geometric mean titer; Nab, neutralizing antibodies; NA, neutralizing antibody.

Evaluations on etiologies of patients on dialysis revealed that diabetic nephropathy, glomerulonephritis, hypertension, nephrosclerosis and nephroangioesclerosis, congenital causes and familial/hereditary causes, polycystic kidney disease, interstitial nephritis, pyelonephritis, urological, ischemic, and secondary causes were reported by these studies. In addition, etiologies that are other than the above mentioned and unknown causes were also reported in these studies. Diabetic nephropathy was one of the leading causes for patients that were eventually on dialysis, constituting 5-46% of these cases (21, 24, 25, 31, 34, 35, 37, 38, 40–43, 46, 48). Similarly, glomerulonephritis was the other cause that was frequently reported, constituting 6.9-47% of causes (21, 24, 25, 31, 34, 35, 37, 38, 42, 43, 46, 48) (**Table 1**).

Hypertensive kidney diseases constituted 4.2-20.8% of causes for patients on dialysis (25, 31, 34, 38, 43, 48), while vascular nephropathy constituted 8-23% of cases (21, 24, 37, 38). Polycystic kidney disease is also frequently present, in a lower proportion, constituting 0-12.0% of these patients (24, 25, 31, 34, 38, 42, 46, 48). Congenital diseases and familial/hereditary cases constituted 1.5-8.3% and 13.1-36% of cases, respectively (21, 35, 37, 38). Interstitial nephritis and pyelonephritis constituted 0-8.3% and 0-13.1% of cases, respectively (21, 25, 31, 34, 37, 38, 42, 46). Urological causes were reported by 3 studies, compositing 0-6.9% of cohorts (24, 34, 42). Other causes less frequently reported include ischemic nephropathy (48), nephrosclerosis (31), nephroangioesclerosis (34) and secondary causes (34). Though various etiologies were reported in these studies, other causes and unknown causes constitute a large proportion of these cases, which were 0-27% and 4.4-52.5%, respectively (21, 24, 25, 31, 34, 37, 38, 42, 43, 46) (**Table 1**).

For patients that underwent KT, diabetic nephropathy constituted 3.7-33% of cases (21, 24, 25, 34, 37, 40, 41, 43, 46), and glomerulonephritis constituted 19.4-43.4% of cases (21, 24, 25, 34, 37, 43, 46). Hypertensive and vascular nephropathy constituted 4.4-7% and 9.7-13% of cases, respectively (24, 25, 41, 43, 46). Congenital and familial/hereditary causes constituted a higher proportion of cases, specifically 5.6-21.2% and 19.1-25% of cases (24, 25, 43). Interstitial nephritis and pyelonephritis constituted a smaller proportion, which were 3.4-10%, and 1.5-1.6%, respectively (24, 25, 43). Urological causes and secondary causes constituted similar proportions, ranging from 5.3% to 6.7%, and from 4.5% to 5.6% of cases (25, 37, 43, 46). Other causes reported included nephorangioesclerosis (3.3%) (37) and polycystic kidney disease 7.1% (46). Similar to that observed in CKD and patients on dialysis, other causes constituted 3.3-36% of cases (21, 24, 25, 37, 43, 46), while unknown causes constituted 4.8-36.6% of cases (21, 24, 25, 37, 43, 46) (**Table 1**).

A total of 31 studies were included in the present systematic review, involving 11,262 participants. Eighteen studies reported age as mean ± standard deviation (SD), ranging from 36.7 ± 11.53 to 74.9 ± 8.4 years of age (19, 22, 25–27, 29–32, 35–37, 40, 42–44, 47, 49) (**Table 1**). Thirteen studies reported age as median, with median value of total cohort available ranging from 49 to 68 years of age (20, 21, 23, 24, 28, 33). The proportion of females ranged from 22.9% to 57.9% (19–49) (**Table 1**).

### 3.2 Vaccinations and measurements

mRNA-based vaccine was utilized in 26 studies (19–21, 23–34, 36–41, 43, 45, 46, 48, 49), adenovirus vector vaccine was applied in 10 studies (22, 24, 34, 35, 40–42, 44, 46, 47), and inactive whole-virus vaccine was utilized in 4 studies (24, 37, 39, 47). In addition, indigenous inactivated adenoviral vector-based vaccine was also utilized in one study (44) (**Table 1**) (**Figure 2**).

**Figure 3:**
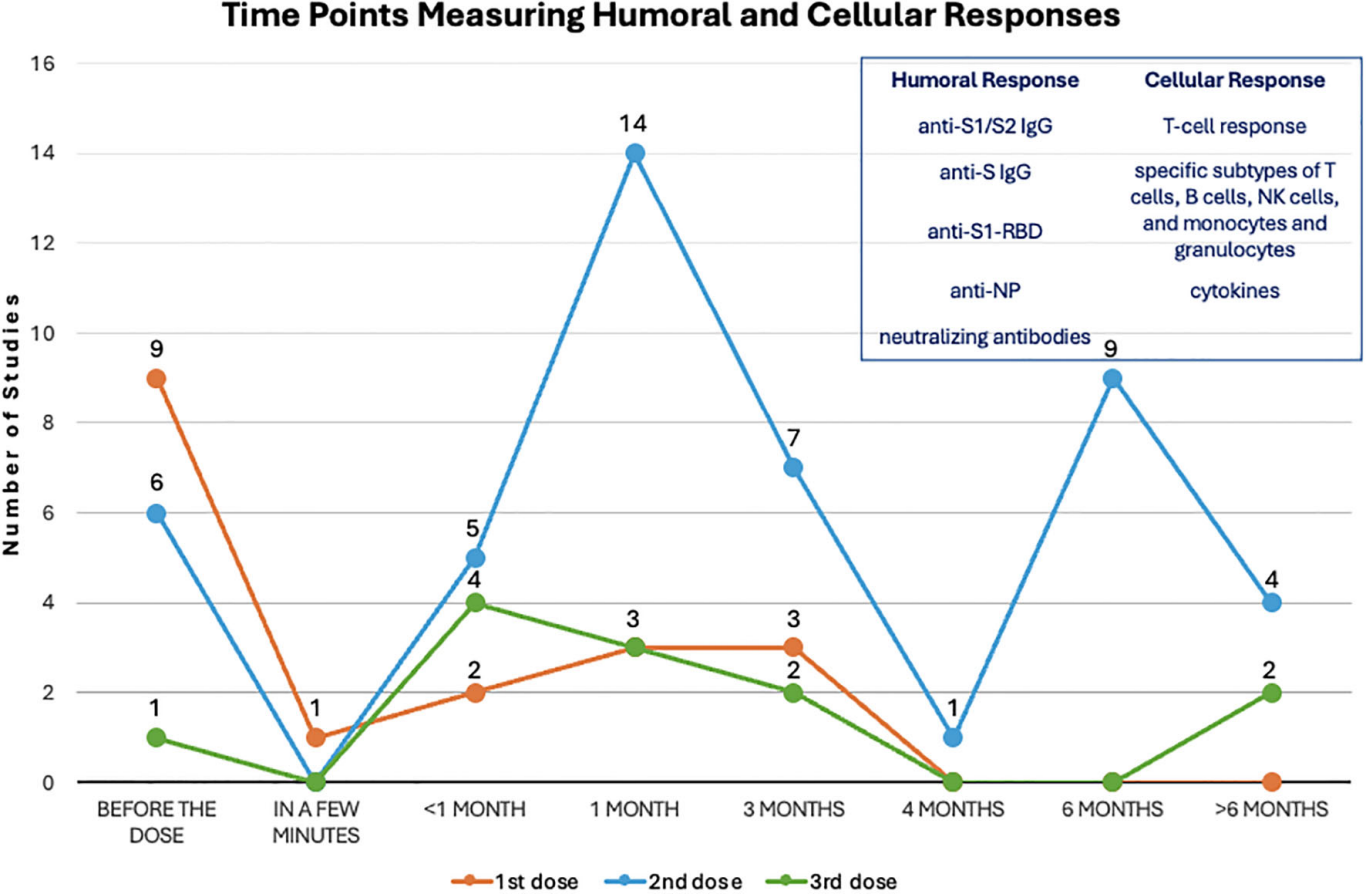
Evaluation time points after each dose. The dots on this line chart indicate the number of studies that evaluate humoral and cellular responses at the specific time point before and after the first (orange), second (blue) and third (green) dose of vaccination. Antibodies used for measuring humoral response and indicators for measuring cellular response are listed in the upper right frame.

Measurement of humoral and/or cellular immune response were performed before the 1^st^ dose (19, 22, 25, 28, 40, 43, 44, 47, 48), or at various time duration after 1^st^ dose, ranging from a few minutes to over 3 months after (19, 21, 22, 26, 42, 44, 48). These assessments have also been performed by studies before 2^nd^ dose (22, 29, 39, 43, 47, 48), less than 1 month (20, 33, 34, 45, 48), at around 1 month (19, 22, 24, 25, 28–30, 35, 36, 39, 40, 43, 44, 46), at around 3 months (20, 24, 26, 31, 34, 38, 47), 4 months (37), 6 months (20, 24, 27, 30, 35, 38, 41, 43, 49), or longer than 6 months (24, 31, 39, 41). If a booster vaccination was introduced, humoral and cellular immune response were evaluated before the 3^rd^ dose (49) and less than 1 month (20, 31, 33, 49), around 1 month (37, 39, 46), 3 months (31, 49), and longer, up to 8–9 months (23, 27) (**Table 1**) (**Figure 3**).

SARS-CoV-2 anti-S1/S2 IgG or anti-S IgG (19–21, 23, 25–28, 31–34, 38, 40, 41, 43, 44, 48, 49), as well as specifically to anti-RBD of the SARS-CoV-2 spike S1 subunit (21–24, 39, 42, 46–48). In addition, SARS-CoV-2 anti-NP was analyzed by 3 studies (23, 47, 48). Three studies did not specify types of antibodies (29, 30, 45). Furthermore, neutralizing antibodies were also assessed by 5 studies (24, 26, 46, 47, 49) (**Table 1**).

SARS-CoV-2 specific T-cell response was measured by 11 studies (25, 26, 28, 31, 34, 35, 37, 43, 45, 47, 49) (**Table 1**). T-cell response was mainly evaluated using the interferon gamma release assay (IGRA) and enzyme-linked immunospot (ELISPOT). Two studies also evaluated specific subtypes of T cells, B cells and NK cells, as well as monocytes and granulocytes (22, 46). Cytokines including IL-4, IL-2, CXCL-10, IL-1β, TNFα, CCL-2, IL-17A, IL-6, IL-10, IFN*γ*, IL-12p70, CXCL-8 (IL-8) and TGFβ1 were also evaluated by one study (45).

### 3.3 Outcomes

#### 3.3.1 Humoral responses

**Figure 4:**
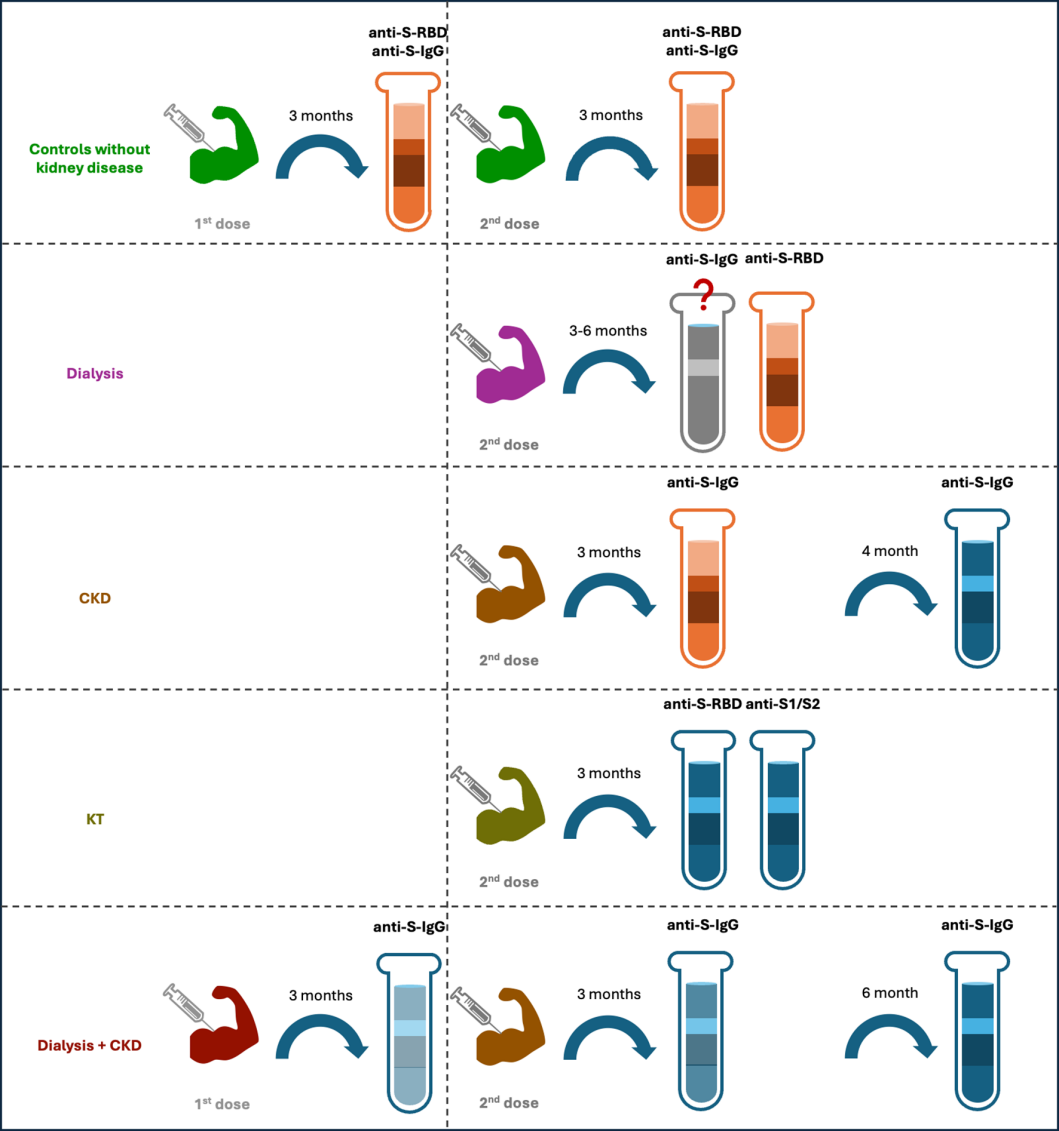
Humoral responses of different cohorts after 1^st^ and 2^nd^ doses of vaccination. For individuals without kidney disease, their anti-S-RBD results were positive three months after the 1^st^ and 2^nd^ doses, and the anti-S-IgG also maintained a high positive rate and at a protective level. For patients with dialysis, some studies have shown a lower titer of anti-S-IgG at 3 and 6 months after the 2^nd^ dose, as compared to the controls, but other studies have shown a similar titer between the two groups. Their anti-S-RBD had a high positive rate after the 2^nd^ dose. For CKD patients, the titer of anti-S-IgG was at similar level of that of the controls at 3 months after the 2^nd^ dose but became lower at 4 months after the dose. For kidney transplant recipients, the positive rate of their anti-S-RBD and anti-S1/S2 were relatively low after the 2^nd^ dose. For a dialysis and CKD mixed cohort, the anti-S-IgG titers decreased significantly at 3 month after the 1^st^ dose, and this rapid decline resulted in a low positive rate at 3 months after the 2^nd^ dose and an even lower rate at 6 months after the 2^nd^ dose. The red color indicates high positive rate, whereas blue color indicates low positive rate, the higher intensity of the red/blue colors, the higher/lower positive rates are.

Comparison of anti-S IgG titers revealed that the antibody titer was lower in patients on HD than in controls with preserved renal functions at 3 weeks (45) and 1 month after 2^nd^ dose (36), and was still lower in patients on dialysis compared to controls without CKD at 3 months and 6 months after 2^nd^ dose by one study (20). In contrast, similar antibody titers between HD and controls without CKD before and at 3 months after 2^nd^ dose (47), and event at 4–8 months after 2^nd^ dose (27) were reported by other studies. Analysis on humoral response after 3^rd^ dose also showed conflicting results, with one study showing a lower anti-S IgG titer in dialysis patients compared to controls without CKD (20), while the other reported a higher level of SARS-CoV-2 IgG in HD than in controls with an estimated glomerular filtration rate ≥ 45ml/min/1773m^2^ at 3 weeks and 3 months after 3^rd^ dose (49). In addition, study on patients on continuous ambulatory peritoneal dialysis (CAPD) also revealed a lower level compared to healthy controls at 3 months after 2^nd^ dose (47). Furthermore, similar decline speed from 1 month to 6 months after 2^nd^ dose between patients on HD and healthy controls was reported by one study (35) (**Table 1**) (**Figure 4**).

The non-response rate in dialysis was reported to be 2.4% in patients on HD at 2 weeks after 2^nd^ dose, in contrast to 0% in controls without kidney failure (32), and 9.09% at 4–8 months after 2^nd^ dose, in contrast to 3.85% in healthy controls (27). However, one study reported a non-response rate as high as 21% in patients on dialysis, in contrast to 0% in healthy controls (19). In addition, the rate was reported to be 0.6% at 2 weeks after 3^rd^ dose, in contrast to 0% in controls without kidney failure (33) (**Table 1**).

Fewer studies compared level of anti-S IgG between CKD and healthy controls directly. Existing studies revealed similar level between the two before and at 3 months after 2^nd^ dose (47), while lower in CKD than in controls at 4 months after 2^nd^ dose (37) (**Table 1**) (**Figure 4**).

Comparison of anti-S IgG titer or median value between CKD and HD showed conflicting results, with one study showing higher anti-S IgG titer at 3 months after 1^st^ dose and higher median value after 2^nd^ dose in CKD compared to patients on HD (21, 30). In contrast, the other study revealed no difference between the two before and at 3 months after 2^nd^ dose (47). Comparison between CKD and KTR revealed a higher titer of anti-S IgG in CKD than in KTR at 3 months after 1^st^ dose (21) (**Table 1**). Few studies compared specific IgG level among different grades of CKDs or between HD and PD. One study revealed similar median value of anti-S IgG between CKD G4 and G5 (30), and one study revealed higher level of IgG in CAPD than in HD at 3 months after 2^nd^ dose (47) (**Table 1**). Furthermore, one study compared IgG level between patients on HD and KTR, and revealed a higher level in patients on HD than in KTR at 4 months after 2^nd^ dose (37) (**Table 1**).

Three studies reported anti-S IgG positivity in proportion following vaccination in different kidney conditions and controls. Specifically, at 15 days after 2^nd^ dose, anti-S IgG was positive in 95% of patients on HD, 93% of patients on PD, 96% of ACKD, 81% of healthy controls, and a noticeable lower proportion of 67% in KTR (34). By 1 month after the last dose, anti-S IgG was positive in 98% of patients on HD, 99% of patients on PD, 100% of patients with CKD, and 79% of KTR (40). By 3 months after 2^nd^ dose, specific IgG positivity was reported in 98% of patients on HD, 100% of patients on PD, 100% of patients with ACKD, 100% of healthy controls, and 75% of KTR (34). At 6 months after 2^nd^ dose, specific IgG positivity was present in 95.1% patients on dialysis, 98.7% patients with CKD G4/5, 100% of controls without kidney disease, and 56.6% KTR (43) (**Table 1**).

Anti-spike RBD IgG revealed a 100% positivity in patients on dialysis, which is significantly higher than a rate of 85.2% in KTR at 3–5 weeks after 3^rd^ dose (46). Similar result on IgG titer was reported by another study showing higher level in patients on dialysis than KTR after full vaccination (1 or 2 doses, depending on vaccination type) through up to 1 year (24) (**Table 1**).

Seroconversion at 3 months after 2^nd^ dose was achieved in 100% patients with CKD, 80.18% of patients on HD, 92.31% of healthy controls and a lower 42.86% of KTR (47). Of note, seroconversion was reported as 0% in CAPD at 3 months after 2^nd^ dose (47), which requires further investigation. The absence antibody response at 1 months after last dose has been suggested to be independently associated with KT (40) (**Table 1**).

#### 3.3.2 Dynamic change in humoral response

In healthy control participants without kidney conditions, the anti-S-RBD was shown positive in 100% of individuals at 1 month after 1^st^ dose (42) and at 3 months after 2^nd^ dose (34). The positivity of anti-S IgG titer decreased by 75.5% at 3 months, while by 88.8% at 6 months after 2^nd^ dose (20). However, seroconversion for anti-S IgG was achieved in 100% of these individuals at 3 months, and still remained in 95% of individuals at 6 months after 2^nd^ dose (20). This is further supported by the fact that anti-S IgG at a protective titer was present in 95% of these individuals at 3 months, and still remained in 70% of individuals at 6 months following 2^nd^ dose (20) (**Table 1**), suggesting a relatively maintenance of humoral response to the vaccines.

Evaluation of anti-RBD alone showed positivity of 94.16% at 1 month after 2^nd^ dose in patients on dialysis, which includes HD and PD (22), and seroconversion for anti-S-RBD was 88.7% at 1 month after 2^nd^ or 3^rd^ doses (29). In addition, anti-S1/S2 IgG was positive in 79% of patients on dialysis at 3 months after vaccination (19), and was present in 94% of patients on dialysis who were previously infected (19) (**Table 1**).

Seropositivity was observed in 88.9% of patients for anti-S-RBD on HD at 1 months after 1^st^ dose (42), and in 64.7% of patients for anti-S at 1 month after 2^nd^ (44). The seroconversion of anti-S IgG reached 96% at 2 weeks (48), and 82.6% at 3 months after 2^nd^ dose, and decreased to 67.9% at 6 months after 2^nd^ dose in patients on dialysis (20), and was 72% after 4^th^ dose in a cohort of combined HD and ND-CKD patients who were previously uninfected (41). However, the titer of anti-S IgG decreased gradually with time. Specifically, a decrease of 82.9% was reported at 3 months after 2^nd^ dose, following by a decrease of 93.03% at 6 months after 2^nd^ dose in patients on dialysis (20). The rapid decrease resulted in achievement of protective titer in 47.7% of patients at 3 months, which subsequently decreased to 23.8% at 6 months after 2^nd^ dose (20), which was less than 50% of that at 1 months (38). Interestingly, the anti-S IgG level in previously infected patients on dialysis at 6 months after recovery was still comparable with infection-naïve patients on dialysis at 1 month after 2^nd^ dose (38), suggesting different humoral responsiveness to different events. When evaluating specifically in patients on HD, anti-S or anti-S-RBD IgG was revealed positive in 95% of patients at 15 days after 2^nd^ dose (34), and to 88-98% of patients at 7–15 weeks after 2^nd^ dose (31, 34), accompanied with a 2.5-fold decrease in level of IgG (34). Then positivity decreased further at 6 months compared to 1 month after 2^nd^ dose (30), while increased to 95% at 3 months following a booster vaccination (31). When observation extended further, one study reported an increase of geometric mean titer (GMT) at 8 months after 2^nd^ dose (39). The seroconversion was 88% for anti-RBD at 2 weeks after 2^nd^ dose (48). The level of neutralizing antibodies was reported to remain unchanged between 8 months after 2^nd^ dose and 1 month after 3^rd^ dose in patients on HD (39), but was reported to increase at 1 month after 3^rd^ dose by another study (37) (**Table 1**).

In patients on PD, positivity of anti-S-RBD IgG was achieved in 93% of these patients at 15 days after 2^nd^ dose, and the proportion increased to 100% at 3 months after 2^nd^ dose (34) (**Table 1**). However, this was accompanied with a 3.75-fold decrease in the level of anti-S IgG at 3 months compared to 15 days after 2^nd^ dose (34).

Few studies evaluated dynamic change of anti-S IgG in other types of kidney conditions. Anti-S-RBD IgG revealed positive in 96% of patients with ACKD at 15 days after 2^nd^ dose (34), which increased to 100% at 3 months after 2^nd^ dose (34). However, a 2.16-fold decrease of anti-S IgG was also revealed at the same time at 3 months, compared to 15 days after 2^nd^ dose (34). Evaluation on CKD also revealed decrease in anti-S IgG at 6 months compared to 1 month after 2^nd^ dose (47), but if administrated with a booster vaccination, could increase at 1 month following (37), peak at 2 months after the dose, and reached a positivity of 93%, with a positivity of 85% for anti-RBD over 9 months following the 3^rd^ dose (23) (**Table 1**).

Analysis on anti-S-RBD IgG revealed a positivity of 67% in KTR at 15 days after 2^nd^ dose, which increased to 75% at 3 months after 2^nd^ dose (34), with anti-S1/S2 IgG positive in 42% of KTR at the same period (19). However, the level of antibody remained low, though increased from 15 days to 3 months after 2^nd^ dose (34). In fact, another study revealed that anti-S IgG positivity was reported to emerge only from 3 months after 2^nd^ dose in KTR (47), and decreased at 6 months compared to 1 month after 2^nd^ dose in KTR (43). However, when administered with a booster dose, larger increase was achieved in KT compared with HD 1 month after (37). The anti-S1/S2 IgG was present in 69% of KTR who were previously infected (19) (**Table 1**).

Age seems to play a role in humoral response to vaccines in patients on dialysis or continuous/intermittent HD. Specifically, age was associated with creased antibody titer (28, 42) and non-respondence (42), but was reported to show no association with production of anti-S IgG at 6 months after 2^nd^ dose (38). Other factors that may be correlated with intermittent HD include previous infection of SARS-CoV-2, neutrophil-to-lymphocyte ratio (NLR), absolute neutrophil count and hemoglobin level (28).

#### 3.3.3 Cellular response

The T-cell response or S-specific T-cell response was revealed in 77.8% of patients with CKD G4/5, 73.3% of patients on dialysis, 17.7% of KTR and 87.5% of controls without kidney disease at 1 month after 2^nd^ dose (43). By 6 months after 2^nd^ dose, the T-cell response was observed in 69.4% of CKD G4/5 (43), 52.6%-67% of patients on dialysis (35, 43), 12.9% of KTR (43) and 48%-75% of controls that are healthy or without kidney disease (35, 43). Interestingly, T cell response was readily detected at baseline in 80% of HD patients, 67% of PD patients, 41% of KT patients, 46% of ACKD patients, while 0% in healthy controls (34) (**Table 1**).

**Figure 5:**
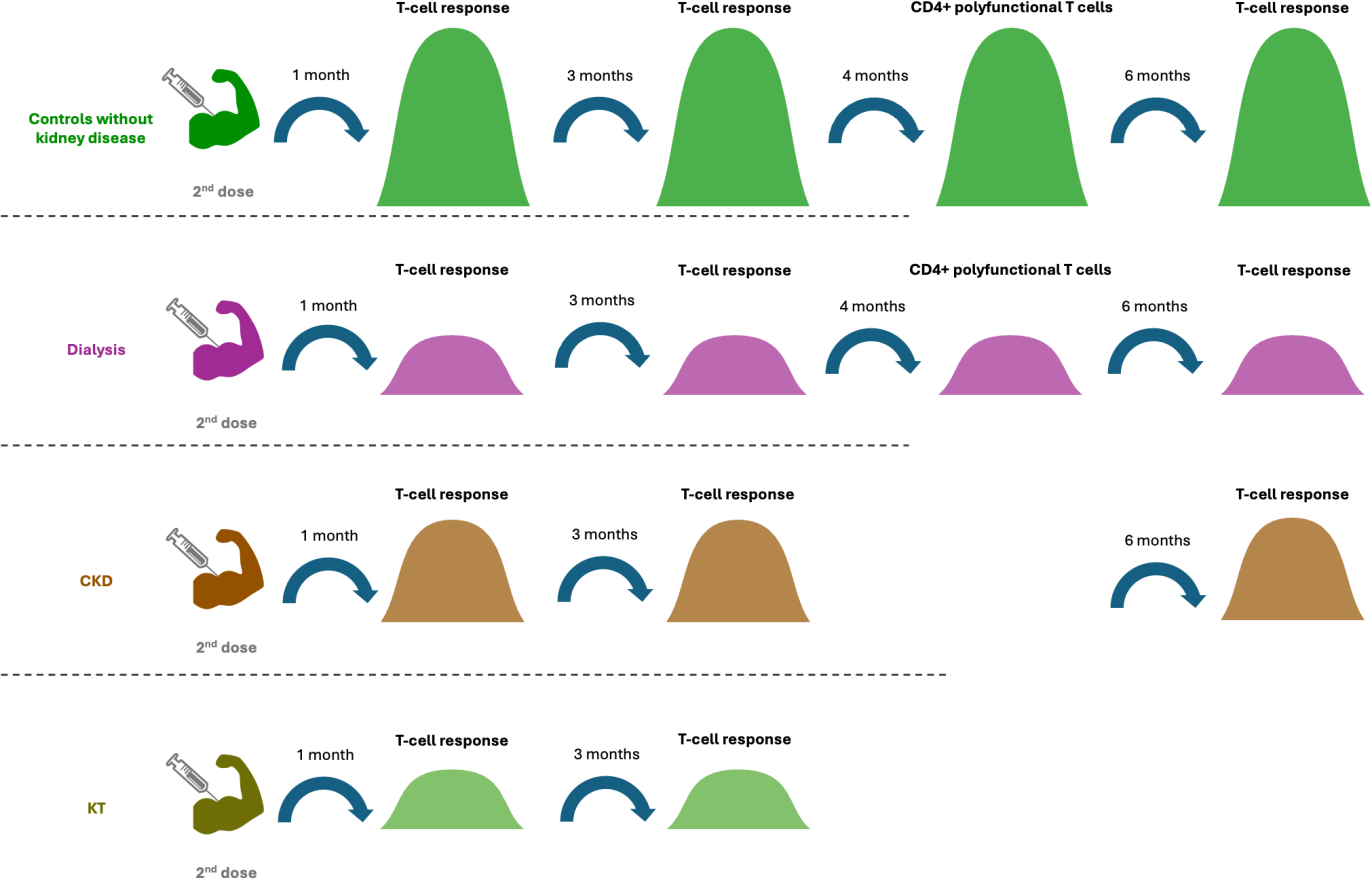
Different cellular responses in different cohorts after the 2^nd^ dose of vaccination. Patients with CKD and dialysis had lower T cell responses from 1 month to 6 months after the 2^nd^ dose compared to controls. At 1 and 3 months after the 2^nd^ dose, the T cell response in CKD was higher than in kidney transplant recipient and dialysis patients. Four months after the 2^nd^ dose, the proportion of the CD4+ polyfunctional T cells in patients with dialysis was lower than that in the control group.

Comparison of T-cell response among different kidney conditions revealed a lower response in CKD and patients on dialysis compared to healthy controls and controls without kidney disease or dialysis at 3 weeks to 6 months after 2^nd^ dose (37, 43, 45, 47), and is further lower in HD that received immunosuppressive therapy (45). In contrast, higher T-cell activity in HD at 3 weeks and 3 months after 3^rd^ dose was reported by one study, compared with controls with an estimated glomerular filtration rate ≥ 45ml/min/1773 m^2^ (49). As expected, S-specific T-cell response was lower in KTR than in controls without kidney disease at 6 months after 2^nd^ dose (43) (**Table 1**) (**Figure 5**).

When evaluated at 4 months after 2^nd^ dose, the proportion of triple positive CD4+ polyfunctional T cells was lower in HD than in healthy controls (37). When evaluation was performed at 1 month after 3^rd^ dose, lower proportion of polyfunctional CD8+ T cells was revealed in CKD, while the proportion of double CD4+ T cells that were positive for CD4, IFN*γ* and IL-2, as well as triple CD4+ T cells that were positive for CD4, IFN*γ*, IL-2 and TNF-α, were higher in KTR compared with healthy controls (37). The proportion of IFN-*γ* (+)-producing CD8+ T cells remained similar among CKD, HD, KT and healthy controls during this time period (37). After the 3^rd^ dose, SARS-CoV-2 specific CD4+ and CD8+ IFN-*γ* responses in memory T cell subsets increased in both CKD and healthy controls (37) (**Table 1**).

Few studies were performed on other immune cell types in other kidney conditions, with one study revealing decrease of proliferative and early active B cells, accompanied with increase of cytotoxin natural killer (cNK) cells in patients on dialysis that did not respond to vaccines at 1 month after 2^nd^ dose (22) (**Table 1**).

Comparison of T-cell response among different kidney conditions revealed a higher response from patients with CKD than in KTRs at 1 month and 3 months after 2^nd^ dose (25, 26) (**Table 1**). In fact, no specific T-cell response was revealed in KTR at 1 month (26). T-cell responsiveness was also higher in CKD than in patients on dialysis (25) or small vessel vasculitis with renal involvement at 1 month and 3 months after 2^nd^ dose, respectively (25, 26).

The proportion of triple positive CD4+ polyfunctional T cells was higher in KTR at 4 months after 2^nd^ dose, and the numbers of double and triple positive CD4+ T cells were higher KTR at 1 month after 3^rd^ dose, compared to HD (37). The responsiveness of T-cells in 1-1.5 month after 2^nd^ dose was associated with level of albumin in CKD patients on intermittent HD (28), and was correlated with level of anti-S IgG at 1 month and 6 months after 2^nd^ dose in CKD and patients on dialysis (43).

#### 3.3.4 Dynamic change in cellular response

Research in healthy control cohort revealed that the proportion of T cell-response increased from 0% at baseline to 67% at 15 days following full vaccination, and then further to 89% following full vaccination (34). In addition, S-specific T-cell response was achieved in 87.5% of controls without kidney disease at 1 month, then decreased significantly to 75% at 6 months after 2^nd^ dose (43). Furthermore, the T-spot count also decreased at 6 months after, compared to 1 month after 2^nd^ dose in healthy controls (35) (**Table 1**).

Though T cell response was present in 46% in patients with ACKD, the response increased to 80% at 15 days, then slightly increased to 89% at 3 months after 2^nd^ dose (34). The T-cell response was present in 77.8% of patients with CKD at 1 month (43), decreased to 72% at 4 months (37), and further to 69.4% at 6 months after 2^nd^ dose (43). An increase of 16% was observed at 1 month after introducing a 3^rd^ dose (37) (**Table 1**).

In patients on dialysis, T-cell activity was detected in 73.3% of the patients at 1 month after 2^nd^ dose (43), decreased significantly to 52.6% at 6 months, and remained at a similar proportion of 55% patients at 6–8 months after 2^nd^ dose (31). The activity was increased again to 85% at 3 weeks after introducing a 3^rd^ dose, then declined to 71% at 3 months after in patients on HD (31). Another study reported T cell response in a considerable proportion of 80% in patients on HD at baseline, which decreased to 70% at 15 days after 2^nd^ dose, followed by an increase to 91% (34). T-cell response was also reported by the same study to be present in 67% of patient on PD at baseline, increased to 93% at 15 days after and achieved 100% at 3 months following full vaccination (34) (**Table 1**).

The T-cell response in KTR varied between studies, with one study reporting 17.7% at 1 month after 2^nd^ dose, which decreased to 12.9% at 6 months after 2^nd^ dose (43). In contrast, the other study revealed presence of T-cell response in 41% of patients, which increased to 84% at 15 days and further to 96% following 2^nd^ dose (34) (**Table 1**).

#### 3.3.5 Correlation between humoral response and cellular response

A few studies evaluated correlations between humoral response and cellular response in patients with kidney conditions. Existing studies revealed co-presence of antibodies and T-cell response in 76.1% of controls with normal or mildly disturbed kidney function and 70.4% of patients with CKD at 1 month after 2^nd^ dose (25), suggesting potential synergy of the two types of immune response The co-presence of the two types of immune response was only present in 54.5% of patients on dialysis (25), but the antibody level was positively correlated with rate of cellular response in patients on HD (28). The correlation between humoral response and cellular response turned to be different in KTRs. One study reported co-presence of the two in 27.9% of KTRs (25) (**Table 1**). However, one study reported that 14.3% of KTRs presented T-cell response in absence of antibody response, while 27.9% of these patients presented antibody response without T-cell response (25), suggesting deficits in specific immune response or a compensation of one type of immune response over another type. The other study reported presence of T-cell response in 75% of patients, but this was only accompanied with humoral response in 49% of patients at 4 months after 2^nd^ dose (37) (**Table 1**).

#### 3.3.6 Potential predictors for non-response

Existing studies suggested that neither eGFR nor urine albumin-creatine ratio (ACR) were associated with antibody levels in CKD that did not need dialysis (23). In contrast, patients that used immunosuppressive treatment were less likely to obtain robust anti-S response (23). In addition, older age seems to play a crucial role in antibody response or antibody decline after vaccination in patients with CKD or on HD (19, 23, 32).

**Figure 6:**
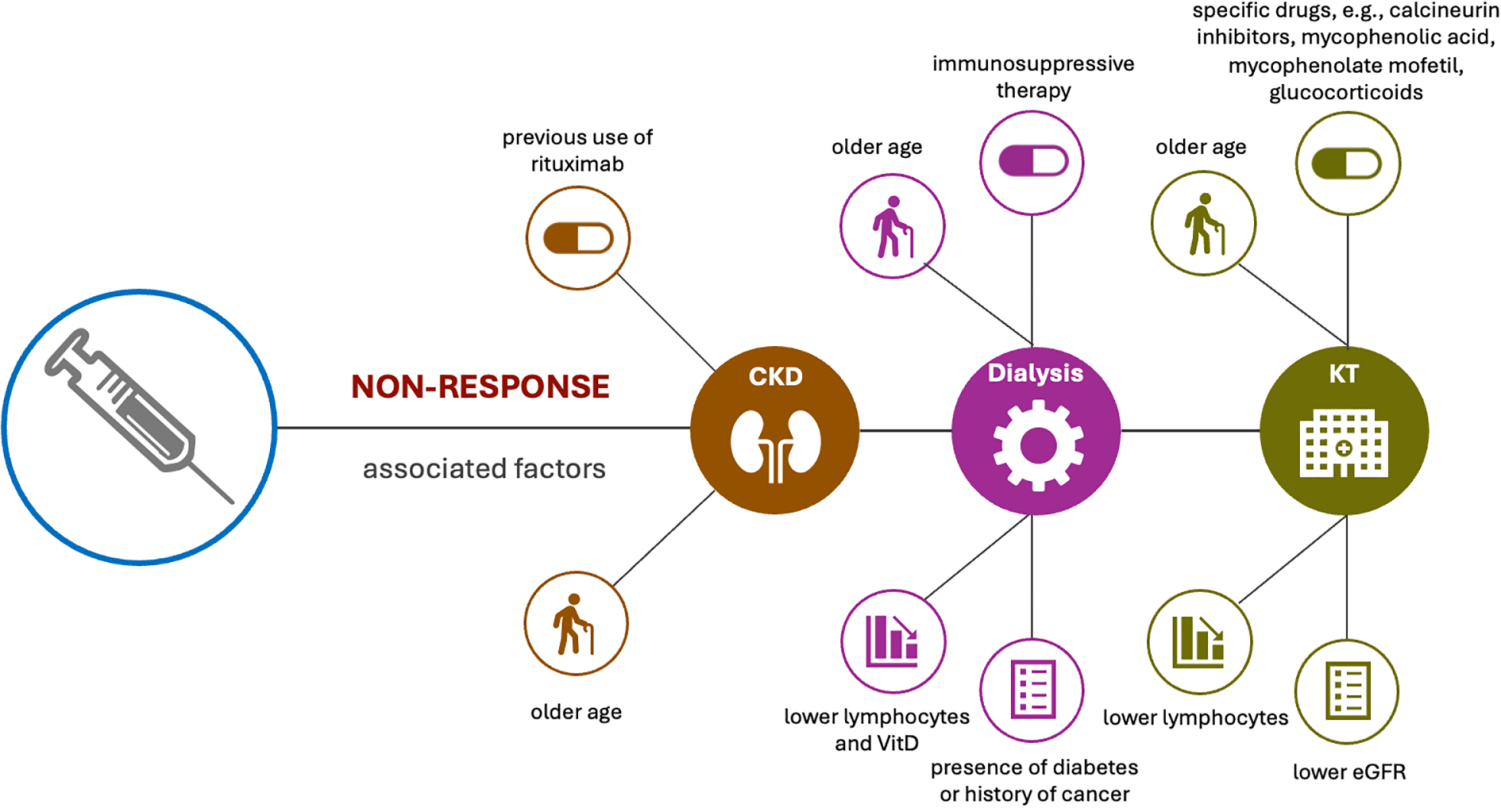
Factors associated with non-response in different types of kidney conditions. Evaluation in CKD revealed a correlation between previous use of rituximab and non-response, and older age also plays a crucial role in antibody response after vaccination in CKD patients. Use of immunosuppressive therapy, older age, presence of diabetes or history of cancer, as well as lower lymphocytes and vitamin D levels have been associated with no-response in patients with dialysis. Factors associated with non-response in KTR tends to be use of specific drugs, older age, lower eGFR and lower lymphocyte.

A few studies have evaluated the factors associated with non-response in different types of kidney conditions. Evaluation in CKD revealed a correlation between previous use of rituximab and non-response (21), while in patient on dialysis or HD, use of immunosuppressive therapy, older age, presence of diabetes or history of cancer, as well as lower lymphocytes and vitamin D have all been associated with non-response following vaccination (19, 29). Factors associated with non-response in KTR tends to be mainly use of specific drugs, such as calcineurin inhibitors, mycophenolic acid, mycophenolate mofetil (MMF) and glucocorticoids (21), though age, lower eGFR and lower lymphocyte have also been associated with non-responder of humoral and cellular immunity after full vaccination (25) (**Figure 6**).

Studies on low-response revealed different factors. In patients on dialysis, modality of dialysis such as HD or PD, high serum ferritin levels, as well as higher monocyte counts were all associated with low-humoral response (19, 46). Study on patients on HD revealed that older age, low BMI, low Cr index, low nPCR and GNRI, as well as administration of steroid administration and complications related to blood disorders were associated with how humoral response after vaccination (32). Analysis in KTRs revealed different factors. Specifically, hypertension, higher calcineurin, use of mTOR inhibitors, higher dose of mycophenolate, as well as lower absolute B-cell counts contributed to low response (46), while higher CD19+ B cell counts were associated with sero-response (46).

### 3.4 Quality of studies

JBI critical appraisal for cohort studies revealed that 7 of the studies were of high quality, 15 were of moderate quality, while 8 were of low quality. Analysis of the one case-control study by NOS revealed that two stars were acquired for the domain of “selection”, one star was acquired for the domain of “comparability”, and two stars were acquired for the domain of “exposure”. The JBI critical appraisal for cohort study and NOS for case-control study is reported in **Table 2**.

**Table 2:** JBI critical appraisal for cohort study and NOS for case-control study.

JBI critical appraisal for cohort study												
Ref	Q1	Q2	Q3	Q4	Q5	Q6	Q7	Q8	Q9	Q10	Q11	Quality
(19)	N	Y	Y	N	NA	N	Y	Y	Y	NA	Y	Low
(20)	N	Y	Y	Y	Y	N	Y	Y	Y	NA	Y	Moderate
(21)	N	Y	Y	U	Y	Y	Y	Y	Y	NA	Y	Moderate
(22)	NA	NA	Y	Y	Y	Y	Y	Y	Y	NA	Y	High
(23)	Y	Y	Y	N	NA	U	Y	Y	Y	NA	Y	Moderate
(24)	N	Y	Y	U	Y	Y	Y	Y	Y	NA	Y	Moderate
(25)	N	Y	Y	Y	Y	U	Y	Y	Y	NA	Y	Moderate
(26)	N	Y	Y	N	NA	U	Y	Y	Y	NA	Y	Low
(27)	N	Y	Y	N	NA	U	Y	Y	Y	NA	Y	Low
(28)	NA	NA	Y	N	NA	N	Y	Y	Y	NA	Y	Moderate
(29)	Y	Y	Y	Y	Y	U	Y	Y	Y	NA	Y	High
(30)	N	Y	Y	N	NA	U	Y	Y	Y	NA	Y	Low
(31)	NA	NA	Y	NA	NA	U	Y	Y	Y	NA	Y	High
(32)	N	Y	Y	N	NA	Y	Y	Y	Y	NA	Y	Moderate
(33)	N	Y	Y	N	NA	Y	Y	Y	Y	NA	Y	Moderate
(34)	N	Y	Y	Y	Y	Y	Y	Y	Y	NA	Y	High
(35)	N	Y	Y	Y	Y	Y	Y	Y	Y	NA	Y	High
(37)	N	Y	Y	N	NA	Y	Y	Y	Y	NA	Y	Moderate
(38)	N	Y	Y	Y	Y	N	Y	Y	Y	NA	Y	Moderate
(39)	NA	NA	Y	N	NA	Y	Y	Y	Y	NA	Y	High
(40)	N	Y	Y	Y	Y	U	Y	Y	Y	NA	Y	Moderate
(41)	N	Y	Y	Y	Y	U	Y	Y	Y	NA	Y	Moderate
(42)	N	Y	Y	Y	Y	Y	Y	Y	Y	NA	Y	High
(43)	N	Y	Y	U	N	Y	Y	Y	Y	NA	Y	Low
(44)	NA	NA	Y	U	N	N	Y	Y	Y	NA	Y	Low
(45)	N	Y	Y	N	NA	Y	Y	Y	Y	NA	Y	Moderate
(46)	N	Y	Y	U	N	Y	Y	Y	Y	NA	Y	Low
(47)	N	Y	Y	U	Y	Y	Y	Y	Y	NA	Y	Moderate
(48)	N	Y	Y	U	Y	N	Y	Y	Y	NA	Y	Low
(49)	N	Y	Y	N	NA	Y	Y	Y	Y	NA	Y	Moderate
NOS for case control study												
Reference	Domain of selection				Domain of comparability				Domain of exposure			
(36)	Two stars				One star				Two stars			

## 4 Discussion

It has been previously reported that CKD patients are at an increased risk for severe outcomes after COVID-19, particularly for those with end stage kidney diseases (ESKD), many of whom have comorbidities now acknowledged as risk factors for severe COVID-19 (2), or who require maintenance of HD. In addition, COVID-19 infection in patients with glomerulonephritis has been reported to result in higher mortality and an increased risk of acute kidney injury compared to controls (50). Long COVID, or post-COVID condition is characterized by a range of symptoms, affecting many organs including kidney. In addition, patients with CKD are considered at increased risk for long COVID (51). Therefore, it is crucial to investigate efficient way such as vaccination to optimize protection of these vulnerable patients from COVID-19 or its severe consequences. Importantly, recent systematic review revealed that administration of COVID-19 vaccines may exert protective as well as therapeutic effects on long COVID (52), highlighting the crucial role vaccination plays in long term management of these patients. For optimal clinical protection after vaccination, both humoral and cellular responses are required. Considering that impaired immune response and immune dysfunction were widely present in CKD with various etiologies, it would be vital to understand whether these features lead to change in humoral and cellular immune response to vaccinations for COVID-19, which were considered an efficient way to reduce spread of infections as well as severity of infections.

Vaccine triggering immune response involves a complex cellular dynamic to activated B-cell response. Antigen and B-cell receptors interaction initiate the early B-cell proliferation. Following the proliferative phase, early B-cells differentiated into the short-lived plasma cells (SLPC), germinal center (GC) cells, and memory B-cells. GCs give more SLPCs, memory Bs and long-lived plasma cells (LLPC) in response to the subsequent antigen stimulation (53). Evaluations with eligible studies that compared humoral response in patients with CKD or CKD requiring dialysis with controls revealed conflicting results, and the extent of decrease in protective antibodies with time after vaccination may vary among diseases and controls. The inferior post-vaccination immunity in dialysis patients (70) could be attributed to immune alterations prevalent in these patients, including skewed Th1/Th2 responses, impaired function of antigen-presenting cells, and susceptibility of B cells to apoptosis (54), leading to a lower likelihood of seroconversion and maintaining protective titers over time (55).

Cellular immunity plays a crucial role in the immune process. CD4+ T cells contribute to protection by supporting isotype switching of B cells, affinity maturation, and clonal proliferation, whereas CD8+ T cells clear virus-infected cells (56, 57). Specifically, CD4+ helper T-cells mediate B-cell-induced antibody production and trigger anti-viral cellular immune responses, whereas CD8+ cytotoxic T-cells can target virus-infected cells and induce their apoptosis. Induction of SARS-CoV-2-specific CD4+ and CD8+ T-cells and higher initial IFNγ production by those cells have been shown to be associated with a milder course of COVID-19 (58). In addition, the T-cell response to SARS-CoV-1 persisted longer after antigen contact than immune protection by antibodies and memory B-cells (59), highlighting the importance of cellular immunity in prevention of COVID-19. Assessment of T-cell immunity revealed great variation in proportion of response in dialysis and ACKD patients. Though studies that only reported T-cell response rate did not seem to reach a conclusion of decrease in CKD at different time points after 2^nd^ dose, a decrease in dialysis patients compared to controls was reported (37, 43, 45, 47). A potential explanation, besides an uremic milieu, could be that the dialysis procedure is associated with diminished immune responsiveness (60). Also, the disturbance of acquired immunity is mainly related to T-lymphocyte and not B-lymphocyte functionality (61). It could also be explained by the use of different cellular assays or different response rate definitions in the various studies. Unfortunately, no attempt was made to discriminate between vaccine-elicited and pre-existing cross-reactive T-cell immunity, an analysis that is far from straightforward. The T-cell response generally precedes the antibody response because of its necessity for priming B cells, and it is maintained for a longer period than the antibody response (62).

Memory T-cells subsets were reported to increase in both CKD and controls, as reported by one study, while mono- and polyfunctional CD4+ and CD8+ T cells was lower in HD and CKD, respectively, compared to controls, highlighting the importance of cellular responses to achieve protection against viral infections and supporting the hypothesis that CD8+ T cells could play an important role in SARS-CoV-2 protection (63).

Of note, KTR consistently showed decreased humoral and cellular response compared to controls, CKD and patients who underwent dialysis. Oral steroids or immunosuppressive drugs were administered to 55.4% of the patients, and cyclosporine and mizoribine were used as immunosuppressive drugs. The use of these medications raised special concerns for KTRs, indicating higher vulnerability of the cohort, highlighting the necessity of an alternative strategy for prevention of COVID-19 in this population.

For optimal clinical protection after vaccination, both humoral and cellular responses are required. Patients with a partial response demonstrated either a humoral response but no cellular response or a cellular response in the absence of a humoral response. Variables associated with nonresponse (both humoral and cellular) were MMF use, lower lymphocyte count and lower eGFR, these variables were also associated with the humoral response alone, indicating that the cellular response is strongly related to the humoral response. However, when we consider the cellular response alone, the use of calcineurin inhibitors seems to be the determining factor for cellular nonresponse and may therefore explain the partial response in these patients. The T-cell response was significantly higher in individuals who seroconverted after a third vaccination, indicating that if an increase in immune response can be detected after repeated vaccination, this will apply to both the humoral and cellular response. This was also reported after a fourth dose. Therefore, additional vaccination doses, administration of heterologous vaccination and monitoring of cellular immunity may be warranted for patients with CKD with or without KT.

The other aspect to consider was kidney vulnerability following vaccination due to dysfunction of the immune system. For instance, IgA nephropathy has a relatively early onset after vaccination and may be associated with rapid immune mechanisms, such as memory recall response and recruitment of cells secreting galactose-deficient IgA1 antibodies. In contrast, the progression of minimal change disease takes a certain amount of time, suggesting the role of cell-mediated immunity (64). It has been reported that many cases of new onset or relapse of glomerulonephritis caused by COVID-19 vaccines were in spontaneous remission or had a good therapeutic response (17). The mechanism underlying podocyte damage after COVID-19 vaccination is hypothesized to involve the expression of permeability factors, such as cytokines and autoantibodies, by stimulating antigen-presenting cells, B cells, and activating T cells, which leads to loss of foot processes and disruption of the glomerular permeation barrier (65). in addition, several COVID-19 infection-related nephritis cases have been reported, and COVID-19 infection is believed to directly cause podocyte damage (66). Evaluations on CKD patients with long COVID-19 revealed significant increase of creatine level compared to controls (67), and the proportion of patients requiring dialysis was also significantly higher (67). In addition, observation over 3 years post infection revealed a slower recovery in CKD patients (68). Pathological observation on animal models of long COVID-19 revealed edema and inflammation of the parenchyma of kidney (69). Together, these results suggested they immune dysfunction may contribute to vulnerability in CKD patients under COVID-19.

There are several limitations for the studies included. Firstly, great heterogeneity was present in etiology of cohorts, severity of diseases, as well as types of vaccinations. Secondly, time points for observation, as well as methods for measurement of humoral and cellular immune response also varied across studies. Studies on carefully screened cohorts with more standardized methods and observation intervals are needed in the future to validate the findings. The systematic review also presented some limitations. Firstly, only studies published in English was included, and studies published in other languages should be included in future studies. Secondly, the present systematic review did not include meta-analysis, which will be desired to evaluate changes in detail in future studies.

## 5 Conclusion

**Figure 7:**
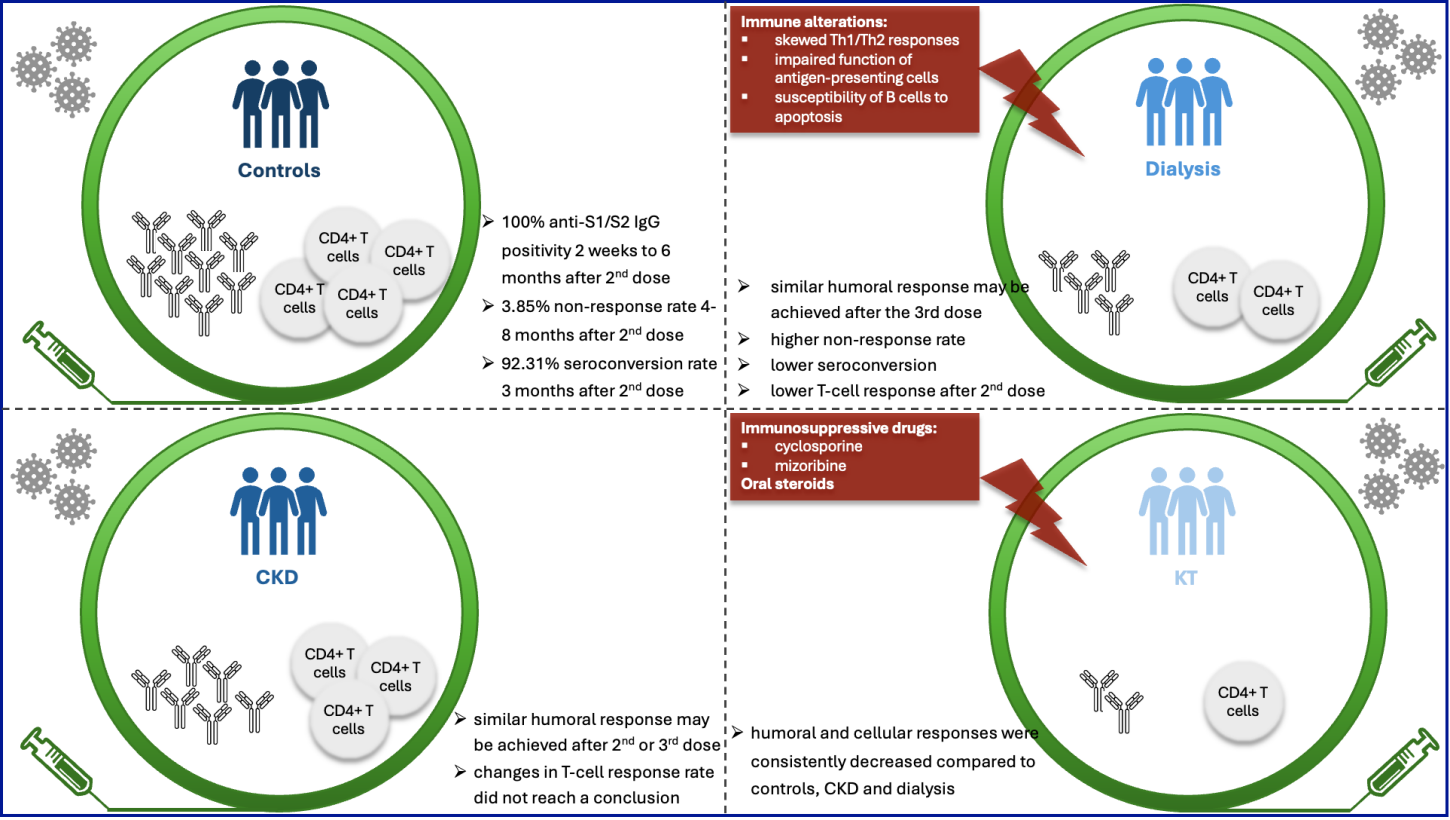
Vaccination provides protection after individuals challenged by coronavirus. For optimal clinical protection after vaccination, both humoral and cellular responses are required. Whether humoral and cellular responses were decreased at different time points after different doses of vaccination in CKD or dialysis patients showed conflicting results, which requires further investigation. Kidney transplantation recipients (KTRs) presented a consistently lower humoral and cellular responses following administration of COVID-19 vaccination across various studies, compared to controls, CKD and dialysis patients.

CKD patients that underwent KT presented a lower humoral and cellular immune response following administration of COVID-19 vaccination. In contrast, whether humoral and cellular response were decreased in CKD or CKD patients who underwent dialysis showed conflicting results and requires further investigation (**Figure 7**). Considering the higher prevalence of kidney manifestations in long COVID-19, understanding the features of change in immune response is crucial for strategy making for management of these patients.

## Data Availability

The original contributions presented in the study are included in the article/supplementary material. Further inquiries can be directed to the corresponding author.
